# ADAR1 and ADAR2 Expression in the Thoracic Aortic Wall Correlates with Aneurysm Severity and Dissection Risk: Insights into A-to-I RNA Editing Dysregulation in Marfan Syndrome-Derived vSMCs

**DOI:** 10.3390/ijms27188250

**Published:** 2026-09-16

**Authors:** Calogera Pisano, Ambra Colopi, Domenico Alessandro Silvestris, Sonia Terriaca, Eugenia Guida, Annamaria Porreca, Adriana Sbrigata, Paolo Niccolò Doronzio, Augusto Orlandi, Susanna Dolci, Piergiorgio La Rosa, Valeriana Cesarini

**Affiliations:** 1 Cardiac Surgery Unit, Department of Precision Medicine in Medical Surgical and Critical Area (Me.Pre.C.C.), University of Palermo, 90127 Palermo, Italy; 2 Department of Research, IRCCS-ISMETT (Mediterranean Institute for Transplantation and Specialized Therapies), 90127 Palermo, Italy; 3 Department of Biomedicine and Prevention, University of Rome Tor Vergata, 00133 Rome, Italy; 4 Department of Biosciences, Biotechnologies and Environment, University of Bari “Aldo Moro”, 70126 Bari, Italy; 5 Department of Human Sciences and Promotion of the Quality of Life, San Raffaele Roma Open University, 00166 Rome, Italy; 6 Unit of Clinical and Molecular Epidemiology, IRCCS San Raffaele Roma, 00163 Rome, Italy; 7 Department of Life Sciences and Public Health, Section of Genomic Medicine, Catholic University of the Sacred Heart, 00168 Rome, Italy; 8 Department of Neuroscience, Section of Human Anatomy, Catholic University of the Sacred Heart, 00168 Rome, Italy; 9 Institute of Genetic and Biomedical Research (IRGB), National Research Council of Italy, Milan Unit, 20138 Milan, Italy; 10 IRCCS Humanitas Research Hospital, Rozzano, 20089 Milan, Italy

**Keywords:** thoracic aortic aneurysm, Marfan syndrome, ADAR1, ADAR2, A-to-I RNA editing, miRNA retargeting, epitranscriptomics, vascular smooth muscle cells

## Abstract

Adenosine-to-inosine (A-to-I) RNA editing, catalyzed by ADAR1 and ADAR2, is an epitranscriptomic mechanism with emerging roles in cardiovascular disease. We investigated ADAR expression, clinical correlates, A-to-I RNA editing signatures, transcriptome and miRNA targetome in thoracic aortic aneurysm (TAA), focusing on Marfan syndrome (MFS). Immunohistochemical analysis of 58 TAA specimens (18 MFS-associated TAA, 19 tricuspid aortic valve-associated TAA (TAV), 21 bicuspid aortic valve-associated TAA (BAV)) showed the highest ADAR1 and ADAR2 in MFS. ADAR2 in vascular smooth muscle cells (vSMCs) correlated positively with aortic diameter across subtypes; ADAR1 inversely correlated with sinotubular junction dimension in MFS. Transcriptomic profiling of MFS-derived vSMC cultures identified 30 differentially expressed genes enriched in immune activation, impaired RTK signaling, and vSMC reprogramming. RNA editing analysis revealed 77 differentially edited sites across 65 genes, predominantly hypoedited in MFS, including four protein-coding recoding events at *PPIL3*, *SLC12A6*, *CTSB*, and *VHL*. miRNA seed-match analysis of 47 significant 3′UTR sites predicted 458 editing-dependent interactions across 36 genes. Only 14/36 genes showed concordant mRNA changes, suggesting that editing-dependent miRNA retargeting acts primarily at the translational level. These findings establish ADAR enzymes as candidates tissue biomarkers of TAA severity and ADAR1 as the principal driver of epitranscriptomic dysregulation in MFS aortopathy.

## 1. Introduction

Thoracic aortic aneurysm (TAA) is a potentially life-threatening condition defined by pathological dilatation of the thoracic aorta, most commonly involving the ascending aorta and aortic root. TAA affects approximately 5.3 per 100,000 individuals per year [1] and is associated with a high risk of aortic dissection and rupture, events carrying an acute mortality exceeding 50% [2,3]. Due to its largely asymptomatic progression, TAA is frequently diagnosed only at the time of a life-threatening complication, underscoring the urgent need for reliable biomarkers of disease progression and improved understanding of its pathogenetic mechanisms [4,5]. Current surgical guidelines rely primarily on aortic diameter thresholds, yet diameter alone is an imperfect predictor of acute events, as a significant proportion of dissections occur in aortas below the surgical threshold [6,7].

TAA arises from a complex interplay of genetic, hemodynamic, and inflammatory factors. The most common heritable causes include mutations in genes encoding structural components of the aortic wall extracellular matrix (ECM) and the contractile apparatus of vascular smooth muscle cells (vSMCs), such as fibrillin-1 (*FBN1*, causing Marfan syndrome), TGF-β receptors (*TGFBR1/2*, causing Loeys-Dietz Syndrome), smooth muscle myosin heavy chain (*MYH11*), and smooth muscle alpha-actin (*ACTA2*) [8,9,10,11]. In non-syndromic TAA, most cases are associated with a bicuspid aortic valve (BAV), which generates abnormal hemodynamic shear stress, or with a normal tricuspid aortic valve (TAV) in the context of hypertension, aging, or connective tissue disorders [12,13]. Despite these distinct etiologies, the histopathological hallmark of TAA is cystic medial necrosis, characterized by vSMC loss, elastic fiber fragmentation, accumulation of proteoglycans, and disruption of the lamellar architecture of the aortic media [14,15]. These changes collectively impair the mechanical integrity and compliance of the aortic wall, progressively increasing the risk of catastrophic failure.

Marfan syndrome (MFS) represents one of the most studied genetic forms of TAA and serves as a paradigmatic model for understanding the molecular pathogenesis of aortic dilatation. MFS is caused by heterozygous loss-of-function mutations in *FBN1*, encoding fibrillin-1, the major structural component of extracellular microfibrils [8]. Beyond its structural role, FBN1 sequesters latent TGF-β complexes within the ECM; *FBN1* mutations therefore result in uncontrolled release and activation of TGF-β, driving a maladaptive cascade that promotes vSMC phenotypic switching, inflammatory cell infiltration, matrix metalloproteinase (MMP) mediated ECM degradation, and progressive aortic root dilatation [16,17]. The vSMC phenotypic switch, from a quiescent contractile state to a proliferative/inflammatory state, is considered a central pathogenetic event in MFS aortopathy and is characterized by downregulation of contractile markers (*MYH11, ACTA2, TAGLN*) and upregulation of inflammatory mediators and matrix remodeling enzymes [18,19]. Inflammatory cytokines, including IL-1beta, IFN-gamma, and TNF-alpha, amplify this process, establishing a feed-forward loop of aortic wall inflammation and structural deterioration [20,21,22].

In parallel with these genetic and inflammatory mechanisms, post-transcriptional regulation has emerged as an additional, largely underexplored layer of complexity in aortic wall biology. Among post-transcriptional modifications, adenosine-to-inosine (A-to-I) RNA editing, catalyzed by the ADAR (Adenosine Deaminase Acting on RNA) family of enzymes, represents the most prevalent form of RNA editing in mammals [23,24]. A-to-I editing converts adenosine to inosine within double-stranded RNA regions, which is interpreted as guanosine by the translational and splicing machinery. This modification can alter protein coding sequences, regulate alternative splicing, modulate miRNA biogenesis and targeting, and affect mRNA stability and nuclear export [24,25]. The ADAR family in humans comprises three members: ADAR1 (p110 and p150 isoforms), ADAR2, and the catalytically inactive ADAR3. ADAR1 and ADAR2 are the principal editing enzymes, with partially overlapping but distinct substrate specificities and expression patterns [26,27].

The role of A-to-I RNA editing in vascular biology has gained increasing attention. Multiple editing sites have been identified within the pre-mRNAs of *MYH11* and *ACTA2*, implicating ADAR enzymes in the post-transcriptional regulation of key vSMC contractile proteins [28]. ADAR1-mediated editing of *Myh11* pre-mRNA leads to its accumulation and downregulation of mature mRNA, suggesting a role in vSMC phenotypic regulation. PDGF-BB, a potent inducer of the vSMC synthetic phenotype, upregulates both ADAR1 isoforms, and ADAR1 knockdown restores contractile protein expression suppressed by PDGF-BB, establishing ADAR1 as a functional mediator of vSMC phenotypic modulation [28]. These effects are recapitulated in vivo in a rat carotid balloon-injury model in which ADAR1 induction coincided with accumulation of unspliced Myh11 and Acta2 pre-mRNAs, while heterozygous ADAR1 knockout markedly attenuated injury-induced neointima formation and preserved contractile marker expression [28]. Furthermore, in abdominal aortic aneurysms (AAA), ADAR1 promotes the stability of matrix metalloproteinases MMP2 and MMP9 mRNAs through interaction with the RNA-binding protein HuR, enhancing metalloproteinase activity and ECM degradation in the altered aortic wall [29]. ADAR1 also contributes to aneurysmal disease through an editing-independent, macrophage-intrinsic mechanism. By binding and promoting degradation of Drosha, it impairs the processing of anti-inflammatory microRNAs targeting NF-κB signaling, thereby sustaining macrophage activation and driving abdominal aortic aneurysm formation in both mouse models and human tissue [30].

ADAR2 shows a distinct expression profile, being highly expressed in healthy arteries and displaying specific vascular editing targets. Chief among these is Filamin A (*FLNA*), an actin-crosslinking protein that interacts with RhoA and ROCK, key regulators of vSMC contraction, and whose ADAR2-mediated editing is essential for normal vascular tone and mechanical homeostasis [31,32]. Reduced *FLNA* editing has been linked to vascular remodeling, elevated blood pressure, and left ventricular hypertrophy [31]. Another ADAR2 target, *IGFBP7*, shows decreased editing in patients with reduced ADAR2 activity and has been associated with cardiomyopathy and aneurysm formation [33]. Together, these findings establish a strong mechanistic link between ADAR-mediated RNA editing and cardiovascular disease, supporting the concept that dysregulation of the epitranscriptome is a pathogenetically relevant feature of aortic wall disease.

Beyond recoding events, A-to-I editing in 3-prime untranslated regions (3′UTR) represents the most abundant class of editing events in the human transcriptome [34,35]. Editing within 3′UTR sequences can create or destroy miRNA binding sites, alter RNA secondary structure, and modulate mRNA stability, constituting a powerful epitranscriptomic mechanism for fine-tuning post-transcriptional gene expression [36,37,38]. The functional interplay between A-to-I editing and miRNA-mediated regulation is particularly relevant in vascular biology, where several miRNAs play established roles in vSMC phenotypic switching, angiogenesis, and aortic wall homeostasis [39,40]. Despite the potential importance of this regulatory axis, its contribution to TAA pathogenesis remains largely unexplored.

In the present study, we aimed to characterize the role of ADAR enzymes and A-to-I RNA editing in the pathogenesis of TAA, with particular focus on MFS. We first analyzed ADAR1 and ADAR2 expression by immunohistochemistry (IHC) in aortic tissue from patients with MFS, BAV-associated TAA, and TAV-associated TAA, correlating enzyme expression with clinicopathological parameters, including aortic dimensions and risk of dissection. We then performed transcriptome-wide differential gene expression analysis and A-to-I RNA editing profiling using publicly available RNA-sequencing data from cultured aortic vSMCs isolated from MFS patients and healthy donors. This cell type-specific approach was chosen given the central role of vSMCs as primary effectors of aortic wall remodeling and as the main cellular substrate of the pathological processes underlying MFS-TAA [41,42]. Our findings reveal a predominantly hypoedited 3′UTR landscape in MFS vSMCs, paradoxically coexisting with ADAR upregulation, and identify specific genes and miRNA-gene regulatory interactions with potential roles in aortic wall degeneration, inflammatory activation, and ECM remodeling.

## 2. Results

### 2.1. ADAR1 and ADAR2 Expression Pattern in TAA Aortic Tissue

**Figure 1:**
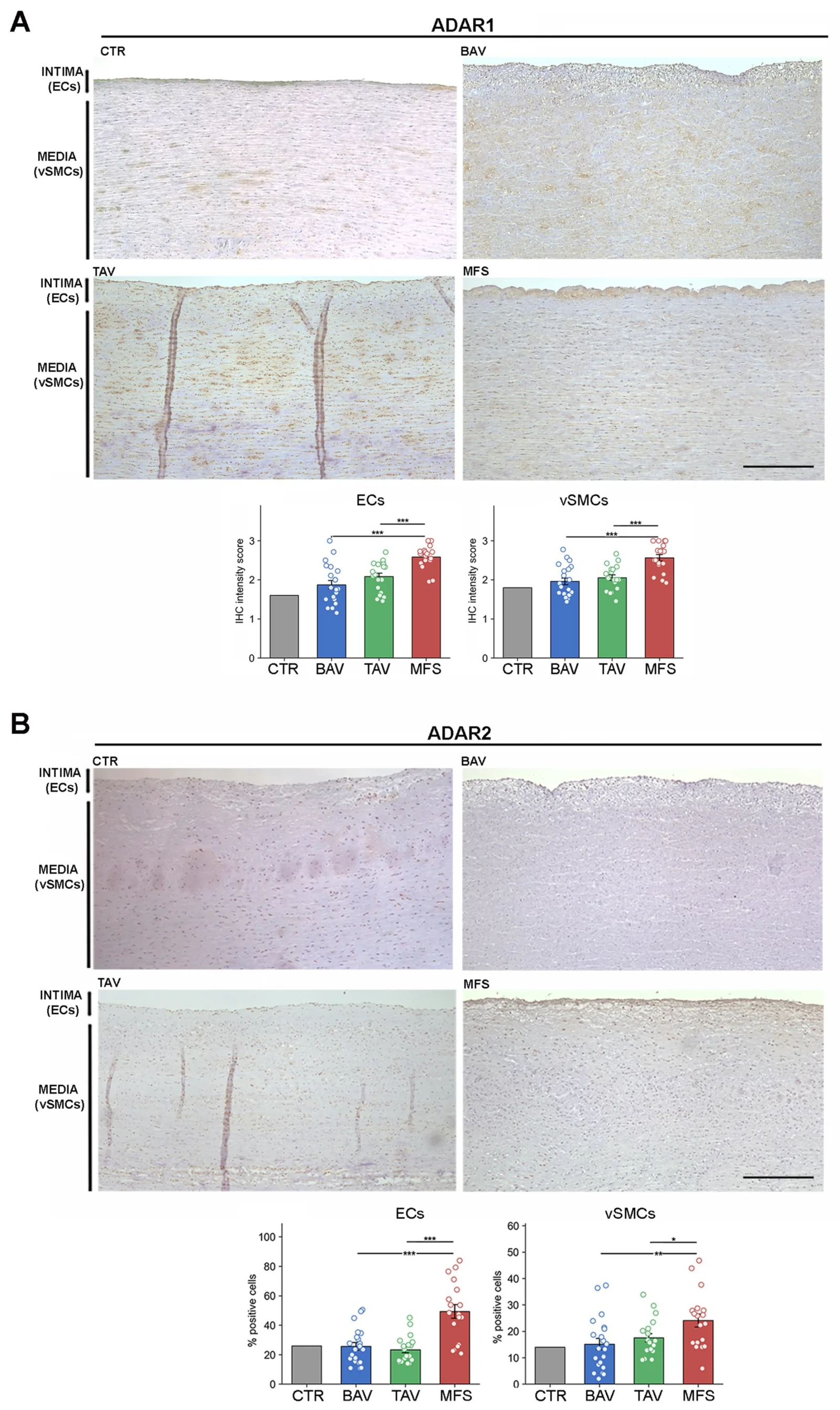
ADAR1 and ADAR2 expression pattern in TAA samples. Representative immunohistochemical images of ADAR1 ((**A**), upper panel) and ADAR2 ((**B**), upper panel) staining in the intimal and medial layers of ascending aortic wall sections from patients with BAV-associated TAA, TAV-associated TAA, MFS-associated TAA, compared to a healthy control aorta from an organ donor. Brown immunoperoxidase staining marks ADAR-positive cells; nuclei are counterstained with hematoxylin, scale bars 200 μm. Quantification of ADAR1 ((**A**), lower panel) protein expression in ECs and vSMCs, scored on a semi-quantitative IHC intensity scale (0–3) and ADAR2 ((**B**), lower panel) in ECs and vSMCs, expressed as percentage of ADAR2-positive cells per field. Data are presented as mean ± SEM. Statistical comparisons were performed by the Kruskal–Wallis test with Dunn’s post-hoc correction. * *p* < 0.05; ** *p* < 0.01; *** *p* < 0.001. BAV, n = 21; TAV, n = 19; MFS, n = 18; Healthy control, n = 1.

We analyzed ADAR1 and ADAR2 protein expression by immunohistochemistry in aortic wall specimens from 58 patients with thoracic aortic aneurysm (TAA), including 18 with MFS, 19 with tricuspid aortic valve (TAV)-associated TAA, and 21 with bicuspid aortic valve (BAV)-associated TAA, as well as one healthy control aorta from a deceased organ donor. Both enzymes were detected in the intimal and medial layers of the aortic wall, with specific staining in both ECs and vSMCs, consistent with the known expression patterns of ADAR enzymes in the vascular compartment [43,44]. In all three TAA subgroups, both ADAR1 and ADAR2 protein levels appeared higher than in the single healthy control aorta available for comparison (Figure 1A,B), with this pattern observed in both the intima and media, suggesting that epitranscriptomic dysregulation may affect multiple cellular compartments of the aneurysmal tissue (Figure 1); given the limited size of the control group (n = 1), this comparison should be regarded as descriptive rather than statistically validated.

ADAR1 expression was particularly elevated in MFS samples compared to TAV and BAV, with MFS specimens exhibiting the highest protein levels in both ECs and vSMCs (Figure 1A). This finding is consistent with the established role of ADAR1 as a downstream effector of pro-inflammatory cytokines, including IL-1beta, IFN-gamma, and TNF-alpha, that are known to be elevated in the MFS aortic wall [20,45]. The pronounced upregulation of ADAR1 in MFS compared to BAV and TAV samples may reflect the particularly intense TGF-β-driven signaling environment of the fibrillin-1-deficient aortic wall [16,17], and is consistent with previous mouse model data in which ADAR1 expression correlated with aneurysm formation [30].

ADAR2 expression also showed significant upregulation in all TAA subgroups, with the highest levels in MFS specimens, particularly in ECs (Figure 1B). Since ADAR2 is classically highly expressed in healthy vascular tissue, where it edits targets such as FLNA and IGFBP7 to maintain vascular homeostasis [31,33], its further upregulation in the aneurysmal wall may represent a compensatory response aimed at sustaining editing-dependent vascular functions in the context of progressive structural deterioration. Together, these data establish that both ADAR1 and ADAR2 are upregulated in the human TAA wall, with MFS representing the most severely affected subgroup, affecting both ECs and vSMCs of the intima and media, respectively.

### 2.2. Correlation Between ADAR1 and ADAR2 Expression and Patients’ Clinical Features

We subsequently investigated whether a correlation exists between the expression levels of ADAR1 and ADAR2 in the altered aortic wall and the patients’ clinical parameters. Demographic and clinical characteristics of the 58 patient cohort are summarized in Supplementary Table S1. MFS patients were significantly younger compared to BAV and TAV groups (median age: MFS TOT 30.0 years vs. BAV 65.0 years and TAV 70.0 years; Kruskal–Wallis *p* < 0.001). Aortic bulb diameter and sinotubular junction dimension also differed significantly across groups (*p* = 0.029 and *p* = 0.034, respectively), whereas ascending aorta diameter and EuroSCORE II did not reach statistical significance (*p* = 0.497 and *p* = 0.602, respectively) (Supplementary Table S1). As already shown in Figure 1, ADAR1 expression in both ECs and SMCs was significantly higher in MFS patients compared to BAV and TAV groups (ADAR1 EC: median 2.7 vs. 1.8 and 2.1; ADAR1 SMC: median 2.7 vs. 1.8 and 2.0; *p* < 0.001 for both). Similarly, ADAR2 expression was markedly elevated in MFS patients, particularly in ECs (ADAR2 EC: median 51.0% in MFS-TAV vs. 23.6% in BAV and 19.1% in TAV; *p* < 0.001), and in vSMCs (ADAR2 SMC: median 24.4% vs. 13.5% and 15.8%; *p* = 0.009) (Supplementary Table S1).

Analysis of classical cardiovascular risk factors and demographic variables revealed no significant associations with ADAR1 or ADAR2 expression levels in either ECs or vSMCs, with one notable exception. Within the BAV and TAV subgroups, where sufficient variability in risk factor distribution allowed statistical testing (Mann–Whitney U test), neither hypertension, smoking, nor diabetes significantly affected ADAR expression (all *p* > 0.05) (Supplementary Table S1). Within the MFS subgroup, this analysis was not feasible for smoking and diabetes due to the absence of internal variability; all MFS patients were smokers, and virtually all were diabetic and hypertensive, consistent with the known syndromic phenotype. Regarding biological sex, no significant difference in ADAR expression between male and female patients was observed in the BAV or TAV groups. However, in the MFS-TAV subgroup, ADAR1 expression in vSMCs was significantly higher in male compared to female patients (*p* = 0.029), suggesting a potential sex-related modulation of ADAR1 activity in the smooth muscle compartment, specifically in the context of MFS (Supplementary Table S1). The independence of ADAR expression from classical cardiovascular risk factors in BAV and TAV patients, combined with the marked upregulation observed specifically in MFS, supports the hypothesis that ADAR dysregulation in the aortic wall is driven by disease-specific molecular mechanisms, most likely the FBN1 mutation-dependent TGF-β dysregulation, rather than by conventional vascular risk factors.

**Figure 2:**
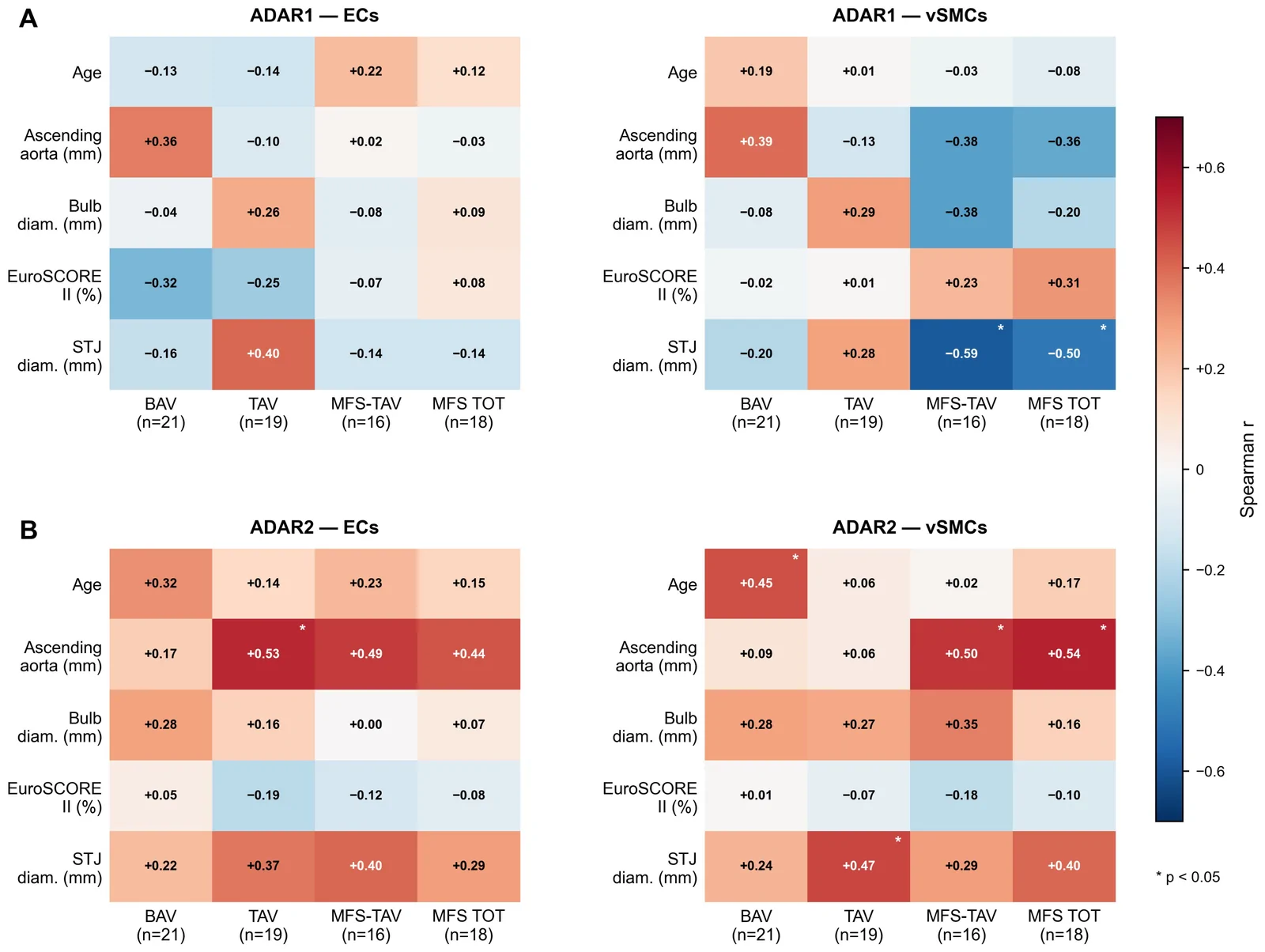
Correlations between ADAR1 and ADAR2 protein expression and aortic clinical parameters across TAA patient subgroups. Each panel shows Spearman rho coefficients for one protein and cell compartment, (**A**) ADAR1 or (**B**) ADAR2 in ECs or vSMCs across four patient subgroups: BAV (n = 21), TAV (n = 19), MFS-TAV (n = 16), and MFS TOT (all MFS patients, n = 18). Color scale ranges from blue (negative correlation) to red (positive correlation). Asterisks (*) indicate statistically significant correlations (*p* < 0.05, Spearman rank-sum test with listwise deletion). The MFS-BAV (n = 2) subgroup was excluded due to insufficient sample size. Asc. aorta, ascending aorta diameter; Bulb diam., aortic bulb diameter; STJ diam., sinotubular junction diameter; EuroSCORE II, European System for Cardiac Operative Risk Evaluation II.

Spearman correlation analysis between ADAR expression and aortic dimensions revealed distinct patterns across patient subgroups (Figure 2, Supplementary Table S2). In MFS patients, ADAR1 expression in vSMCs showed a significant negative correlation with sinotubular junction dimension, both in the MFS-TAV subgroup (r = −0.59, *p* = 0.016), which excludes the 2 patients with BAV congenital defect, and in MFS TOT (r = −0.50, *p* = 0.036), suggesting that higher ADAR1 expression in vSMCs may be associated with less pronounced sinotubular dilation in this specific genetic context. Conversely, ADAR2 expression in vSMCs correlated positively with ascending aorta diameter in both MFS-TAV (r = 0.50, *p* = 0.048) and MFS TOT (r = 0.54, *p* = 0.022), indicating that greater ADAR2 upregulation in the smooth muscle compartment is associated with more advanced aortic dilation in MFS syndrome. In TAV patients, ADAR2 expression in ECs correlated with ascending aorta dimension (r = 0.53, *p* = 0.020), and ADAR2 SMC expression correlated with sinotubular junction diameter (r = 0.47, *p* = 0.042), suggesting a parallel role of ADAR2 upregulation in aortic remodeling among non-syndromic aneurysms as well. In BAV patients, ADAR2 vSMCs expression was positively associated with age (r = 0.45, *p* = 0.039), consistent with a progressive accumulation of ADAR2-driven alterations over time. No significant correlations were found between ADAR1 or ADAR2 expression and EuroSCORE II in any subgroup.

Taken together, these findings indicate that ADAR2 upregulation in vSMCs is consistently associated with greater aortic dilation, particularly in MFS syndrome, whereas ADAR1 overexpression in vSMCs in MFS patients appears to be inversely related to sinotubular junction enlargement. These data support a cell-type- and context-specific role for ADAR enzymes in the structural remodeling of the aortic wall and suggest their potential value as molecular correlates of aneurysm severity.

### 2.3. Transcriptomic Profiling and Functional Enrichment Analysis in MFS-Derived vSMCs

Among the patients analyzed, the MFS subgroup showed the highest ADAR1 and ADAR2 expression levels in both the ECs and vSMC compartments (Figure 1). vSMCs are the predominant cell type of the aortic media and the primary effectors of structural wall remodeling in TAA. The reprogramming of vSMCs is recognized as the central cellular mechanism underlying aneurysm progression and the increased risk of aortic dissection in MFS patients [46]. Furthermore, within the MFS subgroup, ADAR1 expression in vSMCs negatively correlated with sinotubular junction diameter (r = −0.59, *p* = 0.016; Figure 2, Supplementary Table S2), while ADAR2 expression in vSMCs positively correlated with ascending aorta diameter (r = 0.54, *p* = 0.022; Figure 2, Supplementary Table S2), supporting a specific involvement of the vSMC compartment in ADAR-driven aortic remodeling in this patient population. Since A-to-I RNA editing has been linked to vSMC phenotypic switching, with ADAR1 identified as a key regulator of vSMC biology in the context of vascular disease [28], we decided to explore the transcriptomic profile and the A-to-I editing signature of vSMCs isolated from the aneurysmal thoracic aortic wall of MFS patients. We used publicly available RNA-sequencing data from the GEO dataset GSE128101 [47], which includes RNA-seq profiles of cultured aortic vSMCs derived from 3 male MFS patients who underwent aortic root surgery, and from 3 healthy organ-donor controls. The use of RNA-sequencing data from isolated aortic SMC cultures (GSE128101) provides a cell-type-specific perspective on the molecular dysregulation underlying MFS aortopathy, reducing the confounding effects of cellular heterogeneity inherent to whole-tissue RNA extracts. In this context, the transcriptomic and RNA editing results described below should be interpreted as reflecting specifically the vSMC compartment of the MFS aortic wall.

**Figure 3:**
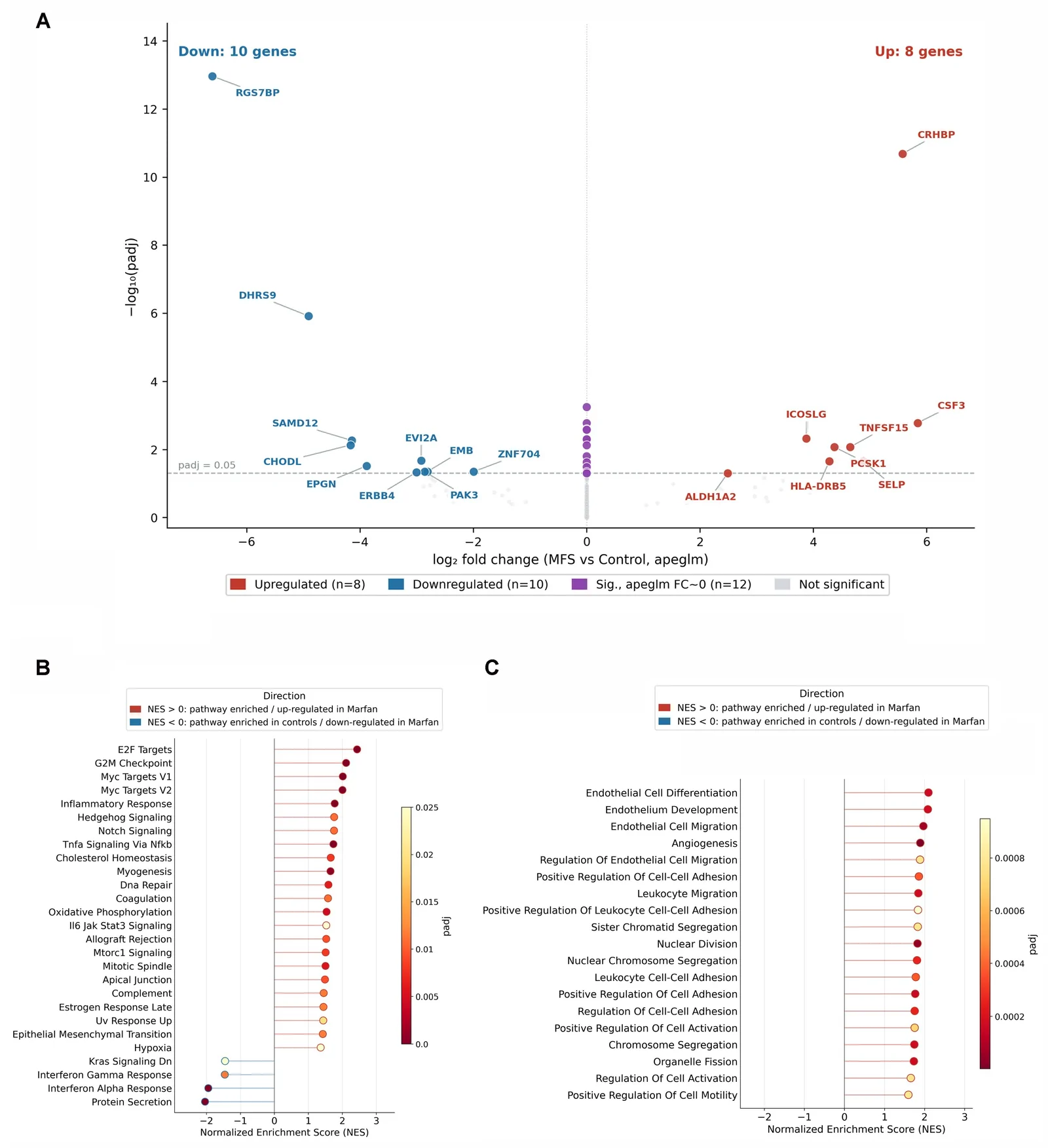
Transcriptomic analysis of MFS-derived vSMCs. (**A**) Volcano plot of differential gene expression between MFS and control vSMCs. The *x*-axis shows the apeglm-shrunken log_2_ fold change (MFS vs. Control) and the *y*-axis shows −log_10_(adjusted *p*-value). Red dots indicate significantly upregulated genes (padj < 0.05, log_2_FC > 0; n = 8), blue dots indicate significantly downregulated genes (padj < 0.05, log_2_FC < 0; n = 10), and purple dots indicate genes with padj < 0.05 but log_2_FC shrunk to ~0 by apeglm (n = 12). Grey dots represent non-significant genes. The dashed horizontal line marks the significance threshold (padj = 0.05). Gene labels are shown for all significantly up- and downregulated genes. Gene Set Enrichment Analysis (GSEA) dot plots showing significantly enriched gene sets in MFS vs. control vSMCs in terms of (**B**) MSigDB Hallmark gene sets (padj < 0.05; n = 27) and (**C**) Gene Ontology Biological Process (GO:BP) terms (padj < 0.001; n = 19). The *x*-axis represents the Normalized Enrichment Score (NES); positive NES indicates pathway enrichment in MFS, negative NES indicates enrichment in controls. Dot color reflects statistical significance (padj), with darker red indicating greater significance. Gene sets are sorted by ascending NES.

Differential expression analysis (DESeq2, apeglm shrinkage; padj < 0.05) identified 30 differentially expressed genes (DEGs): 27 protein-coding and three lncRNAs (*FAM106A*, *EPHA5-AS1*, *TTTY16*). Eight genes were upregulated, *CSF3* (log2FC = +5.84), *CRHBP* (+5.58), *SELP* (+4.89), *TNFSF15* (+4.65), *PCSK1* (+4.37), *HLA-DRB5* (+4.28), *ICOSLG* (+3.88), *ALDH1A2* (+2.49), and ten were downregulated: *RGS7BP* (log2FC = −6.61), *DHRS9* (−4.91), *CHODL* (−4.17), *SAMD12* (−4.14), *EPGN* (−3.88), *ERBB4* (−3.00), *EVI2A* (−2.92), *PAK3* (−2.86), *EMB* (−2.81), *ZNF704* (−1.99). The remaining 12 DEGs showed fold changes shrunk to near zero by apeglm, indicating low-effect-size, high-precision signals (Figure 3A, Supplementary Table S3).

Among the upregulated genes, several have established roles in vascular inflammation. *TNFSF15* (TL1A) regulates vSMC phenotypic switching between contractile and synthetic states [48]; P-selectin (*SELP*) mediates vascular inflammation with documented involvement in aortic aneurysm pathophysiology [49]; *ICOSLG* may facilitate interactions between vSMCs and adventitial lymphocytes under inflammatory conditions [50]. *ALDH1A2*, involved in retinoic acid biosynthesis, has not been previously reported in MFS vSMCs and may represent a novel link between retinoid signaling and vSMC dysfunction. Among the downregulated genes, *ERBB4*, a receptor tyrosine kinase activated by neuregulin-1 and a candidate gene in BAV-associated aortopathy [51], and *PAK3*, a kinase with established functions in vSMC migration and survival [52], are of particular functional interest.

Gene Set Enrichment Analysis (GSEA) against the MSigDB Hallmark collection identified 27 significantly enriched gene sets (padj < 0.05), organizing into three biologically coherent clusters (Figure 3B,C). The most strongly enriched upregulated cluster comprised cell cycle and proliferation gene sets, e2f_targets (NES = 2.44), g2m_checkpoint (NES = 2.11), myc_targets_v1 (NES = 2.02), myc_targets_v2 (NES = 2.01), mitotic_spindle (NES = 1.51), and dna_repair (NES = 1.59), with leading-edge genes including *CDK1, AURKB, PLK1, MKI67, BIRC5,* and *MAD2L1*, consistent with a shift toward a proliferative synthetic vSMC phenotype driven by TGF-β dysregulation and fibrillin-1 deficiency [16,17]. A second cluster encompassed inflammatory signaling, including inflammatory_response (NES = 1.78; *CSF3, TNFSF15, ICAM1, IL1B, IL6*), tnfa_signaling_via_nfkb (NES = 1.74), il6_jak_stat3_signaling (NES = 1.53), and allograft_rejection (NES = 1.53), reflecting the chronic pro-inflammatory environment of the fibrillin-1-deficient aortic wall [20]. A third cluster indicated broader phenotypic plasticity, with enrichment of notch_signaling (NES = 1.76; *NOTCH1, NOTCH3, JAG1, HES1*), myogenesis (NES = 1.65), epithelial_mesenchymal_transition (NES = 1.43), and metabolic gene sets (cholesterol_homeostasis, NES = 1.66; oxidative_phosphorylation, NES = 1.54), suggesting multi-directional vSMC transdifferentiation and metabolic reprogramming.

Among the downregulated gene sets, protein_secretion showed the strongest negative enrichment (NES = −2.04, padj = 5.88 × 10^−5^; *COG2, SNAP23, CLTC, RAB5A, COPB2*), indicating impaired vesicular trafficking and secretory function, potentially reflecting a diversion of cellular resources away from extracellular matrix production. Strikingly, both interferon_alpha_response (NES = −1.94) and interferon_gamma_response (NES = −1.46) were negatively enriched, with *ADAR1* (encoded by the *ADAR* gene) in the leading edge of both gene sets (Figure 3B). As *ADAR1* is a well-established interferon-stimulated gene, its transcriptional downregulation in cultured MFS vSMCs, despite protein-level upregulation in tissue, likely reflects the loss of the in vivo inflammatory cytokine milieu ex vivo, and highlights the importance of interpreting cell culture transcriptomics in light of the original tissue context.

Complementary GSEA against GO Biological Process terms confirmed these findings (Figure 3C), with the most significantly upregulated terms including endothelial cell differentiation (NES = 2.10), endothelial cell migration (NES = 1.97), angiogenesis (NES = 1.89), leukocyte migration (NES = 1.85), nuclear division (NES = 1.83), and chromosome segregation (NES = 1.75), collectively depicting MFS vSMCs as proliferating, inflammatory, and phenotypically plastic cells undergoing broad transcriptional reprogramming toward a maladaptive synthetic state.

### 2.4. A-to-I RNA Editing Landscape in MFS-Derived vSMCs

**Figure 4:**
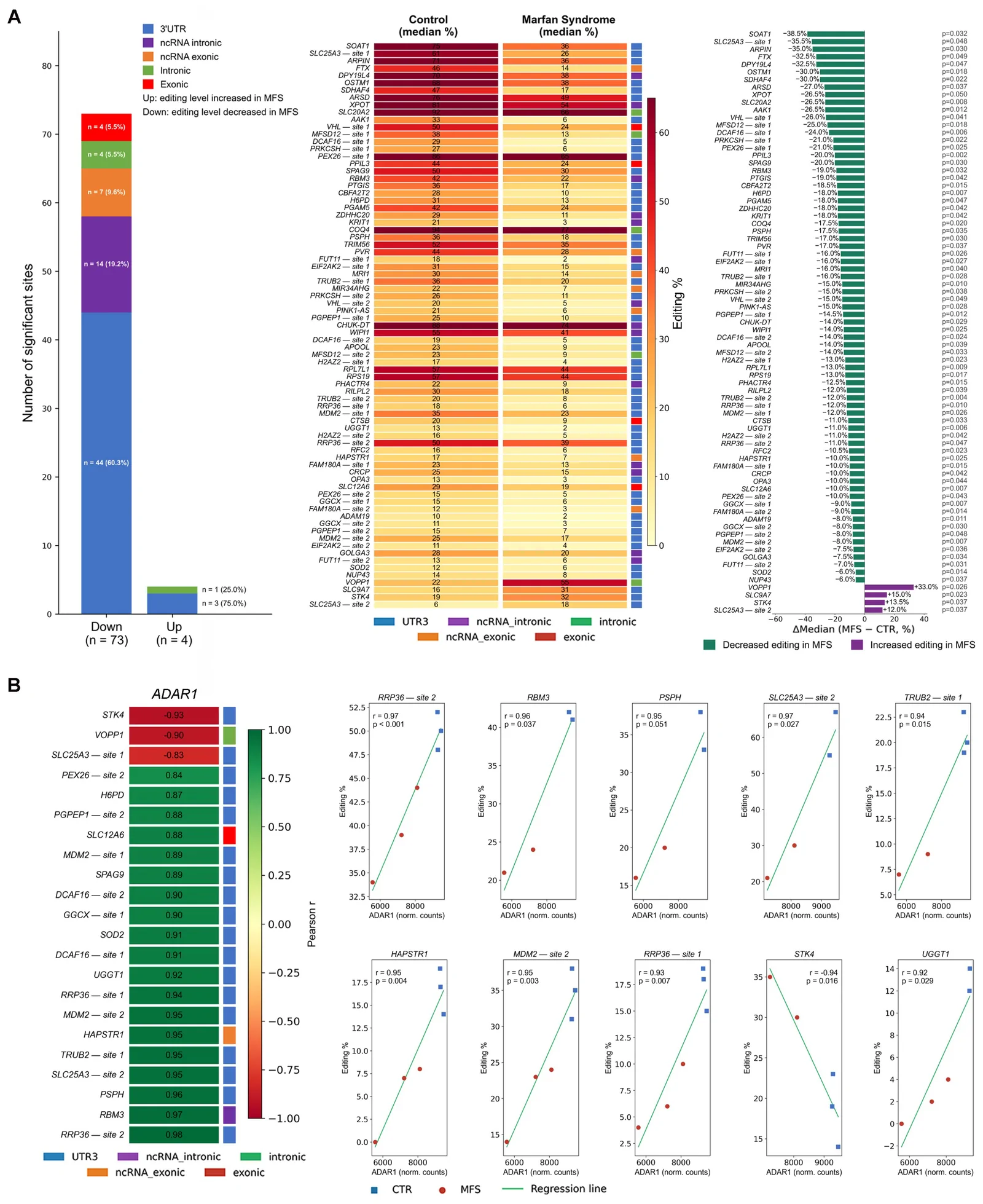
A-to-I RNA editing in MFS-derived vSMCs. (**A**) Left panel, distribution of significant A-to-I RNA editing sites by genomic region and direction of change in MFS syndrome-derived vSMCs. Significant editing sites (*p* < 0.05, Wilcoxon rank-sum test) are grouped by direction of change relative to controls: hypoedited (decreased editing in MFS, n = 73) and hyperedited (increased editing in MFS, n = 4). Bar segments indicate the number and proportion of sites in each genomic region: 3′UTR (blue), ncRNA intronic (purple), ncRNA exonic (orange), intronic (green), and exonic (red). Percentages refer to the proportion within each group. Middle panel, heatmap showing the median editing level (%) at 77 significant differentially edited sites (*p* < 0.05, |ΔMedian| ≥ 10%) in control (CTR) and MFS syndrome (MFS) vSMCs. Colored squares on the right indicate the genomic region of each site (blue: 3′UTR; purple: ncRNA intronic; green: intronic; orange: ncRNA exonic; red: exonic). Right panel, horizontal bar chart showing the ΔMedian editing level (MFS − CTR, %) for each site. Purple bars indicate sites with increased editing in MFS (hyperediting); teal bars indicate sites with decreased editing in MFS (hypoediting). Adjusted *p*-values are shown for each site. (**B**) Left panel, heatmap of Pearson correlation coefficients (r) between per-sample editing level and *ADAR1* (*ADAR* gene) mRNA expression (DESeq2 normalized counts, GSE128101) for the 22 sites showing statistically significant *ADAR1* correlation (*p* < 0.05), ranked by decreasing r. Colored squares indicate the genomic region as in panel A. Right panel, scatter plots of per-sample editing level (%) versus *ADAR1* normalized counts for the top 10 positively correlated sites. Blue squares: control samples; red circles: MFS samples. Green line: linear regression. Pearson r and *p*-value are indicated for each site.

To identify the A-to-I RNA editing landscape in MFS-derived vSMCs, we performed RNA editing analysis on the GSE128101 dataset, and we identified a total of 77 significant A-to-I RNA editing sites across 65 genes (*p* < 0.05, |ΔMedian| ≥ 10%), distributed across multiple genomic regions, 47 in 3′UTR, 14 in ncRNA intronic, 5 in intronic, 7 in ncRNA exonic, and 4 in exonic regions. Of these, 73 sites showed reduced editing in MFS vSMCs compared to control, while 4 sites exhibited increased editing (Figure 4A, Supplementary Table S4).

Notably, among the 77 significant sites, four were located within protein-coding exons, representing the only recoding (A-to-I) events that directly alter the amino acid sequence or codon usage of the encoded protein. All four exonic sites were down-edited in MFS vSMCs relative to controls. Peptidylprolyl Isomerase Like 3 (PPIL3), a cyclophilin-domain protein associated with the spliceosome C-complex, harboured a nonsynonymous site (c.A175G; p.S59G) previously catalogued among conserved A-to-I recoding events in human cardiovascular tissues [53], with editing levels of 44% in CTR versus 24% in MFS (ΔE = −20%, *p* = 0.0018). The edited glycine replaces a phosphorylatable serine, potentially altering PPIL3 interaction with splicing regulators; reduced editing in MFS may therefore shift the balance toward the serine-containing isoform and affect spliceosome dynamics. *SLC12A6*, encoding the K–Cl cotransporter KCC3, showed a nonsynonymous editing in exon 26 (c.A3422G; p.H1141R) not previously reported, with editing levels decreasing from 29% (CTR) to 19% (MFS) (ΔE = −10%, *p* = 0.0072). KCC3 regulates cell volume and ion homeostasis in vSMCs; the histidine-to-arginine substitution at position 1141 introduces a positively charged residue in the cytoplasmic C-terminal domain, and its reduced occurrence in MFS vSMCs may affect cotransporter activity or regulatory interactions. Cathepsin B (CTSB), a lysosomal cysteine protease involved in extracellular matrix remodelling and previously implicated in aortic aneurysm pathophysiology [54], exhibited nonsynonymous editing at two positions (p.R31G and p.R268G), with editing decreasing from 20% (CTR) to 9% (MFS) (ΔE = −11%, *p* = 0.033). Notably, *CTSB* mRNA editing by ADAR1 has been previously described as part of an autoregulatory feedback loop. S-nitrosylation of the CTSB protein promotes ADAR1 recruitment to its own transcript via the ADD1/MATR3 axis, stabilising the mRNA and sustaining CTSB protein levels [55]. Both arginine-to-glycine substitutions remove positively charged residues from functionally critical regions of the protease domain, and their reduced frequency in MFS may favour the unedited, arginine-bearing form with potentially altered catalytic or substrate-binding properties; moreover, the hypoediting of *CTSB* observed in MFS vSMCs may disrupt this autoregulatory loop, with potential consequences for *CTSB* mRNA stability and protein expression. Finally, Von Hippel-Lindau tumour suppressor (VHL), the E3 ubiquitin ligase adaptor that targets HIF-1α for proteasomal degradation and is central to the cellular hypoxia response, carried a synonymous editing event in exon 2 (c.A372G; p.Q124Q) not previously reported with the highest differential observed across all exonic sites (CTR 50% versus MFS 24%, ΔE = −26%, *p* = 0.041). Although this edit does not change the encoded glutamine, synonymous recoding events can influence mRNA folding, translational kinetics, and alternative splicing; the pronounced hypoediting at this site in MFS vSMCs raises the possibility of altered *VHL* transcript processing and, consequently, dysregulation of hypoxia-inducible signalling in the diseased aortic wall.

To determine whether the differentially edited sites are preferentially regulated by ADAR1 or ADAR2, we extended the per-site inferential analysis by correlating, for each of the 77 editing sites individually, the per-sample editing level with the per-sample mRNA expression of *ADAR1* and *ADAR2*. ADAR1 mRNA showed statistically significant positive correlations (*p* < 0.05) with 19 of the 77 sites (Figure 4B, Supplementary Table S5). The strongest positive correlations were observed for *RRP36*, site 2 (r = 0.980, *p* < 0.001), *RBM3* (r = 0.970, *p* = 0.030), *PSPH* (r = 0.960, *p* = 0.040), and SLC25A3, site 2 (r = 0.955, *p* = 0.045). Three sites showed significant negative ADAR1 correlations: *STK4* (r = −0.928, *p* = 0.023), *VOPP1* (r = −0.903, *p* = 0.036), and SLC25A3, site 1 (r = −0.826, *p* = 0.043), all of which correspond to sites with increased editing in MFS (Supplementary Table S4). In contrast, *ADAR2* mRNA did not show statistically significant correlations with editing at any of the 77 sites (all *p* > 0.05), indicating that the editing landscape in MFS vSMCs is predominantly driven by ADAR1. The absence of significant *ADAR2* correlations is consistent with the high variability in *ADAR2* expression across individual samples (normalized counts range: 159–891), which limits statistical power to detect site-specific associations. (Supplementary Table S5).

### 2.5. A-to-I RNA Editing at 3′UTR Sites Remodels the miRNA Targetome in MFS-Derived vSMCs

miRNA target recognition occurs through base-pairing between the miRNA seed region (nucleotides 2–8 from the 5-prime end of the mature miRNA) and a complementary sequence in the target 3′UTR. Four canonical seed match types are defined in decreasing order of functional strength: the 8mer site, the 7mer-m8 site, the 7mer-1a site, and the 6mer site. The 8mer and 7mer-m8 types are associated with the strongest target repression, while 6mer matches are less predictive of functional regulation [56,57].

A-to-I editing within 3′UTR sequences constitutes a direct mechanism of miRNA target site rewiring. By converting adenosine to inosine (read as guanosine by the cellular machinery), editing can create or destroy complementarity between miRNA seed regions and their target sequences, thereby redirecting which miRNAs repress a given transcript, a phenomenon termed miRNA retargeting [58,59]. This editing-dependent retargeting operates at the level of individual seed match sequences (6mer, 7mer, 8mer), where even a single nucleotide change can abolish (lose) or generate (gain) a functional miRNA binding site, with downstream consequences for transcript stability and translational efficiency [38].

Some miRNAs recognize and bind the 3′UTR sequence only when a given site is edited, while others bind only when the site is unedited. In MFS, where the editing level is globally reduced at these loci, the unedited adenosine form prevails. Consequently, miRNAs that require the edited sequence for binding become less active, while miRNAs that bind the unedited sequence become more active. This shift in the miRNA binding landscape directly influences the post-transcriptional regulation of the target gene, resulting in a disease-specific rewiring of the post-transcriptional regulatory network in MFS aortic vSMCs.

**Figure 5:**
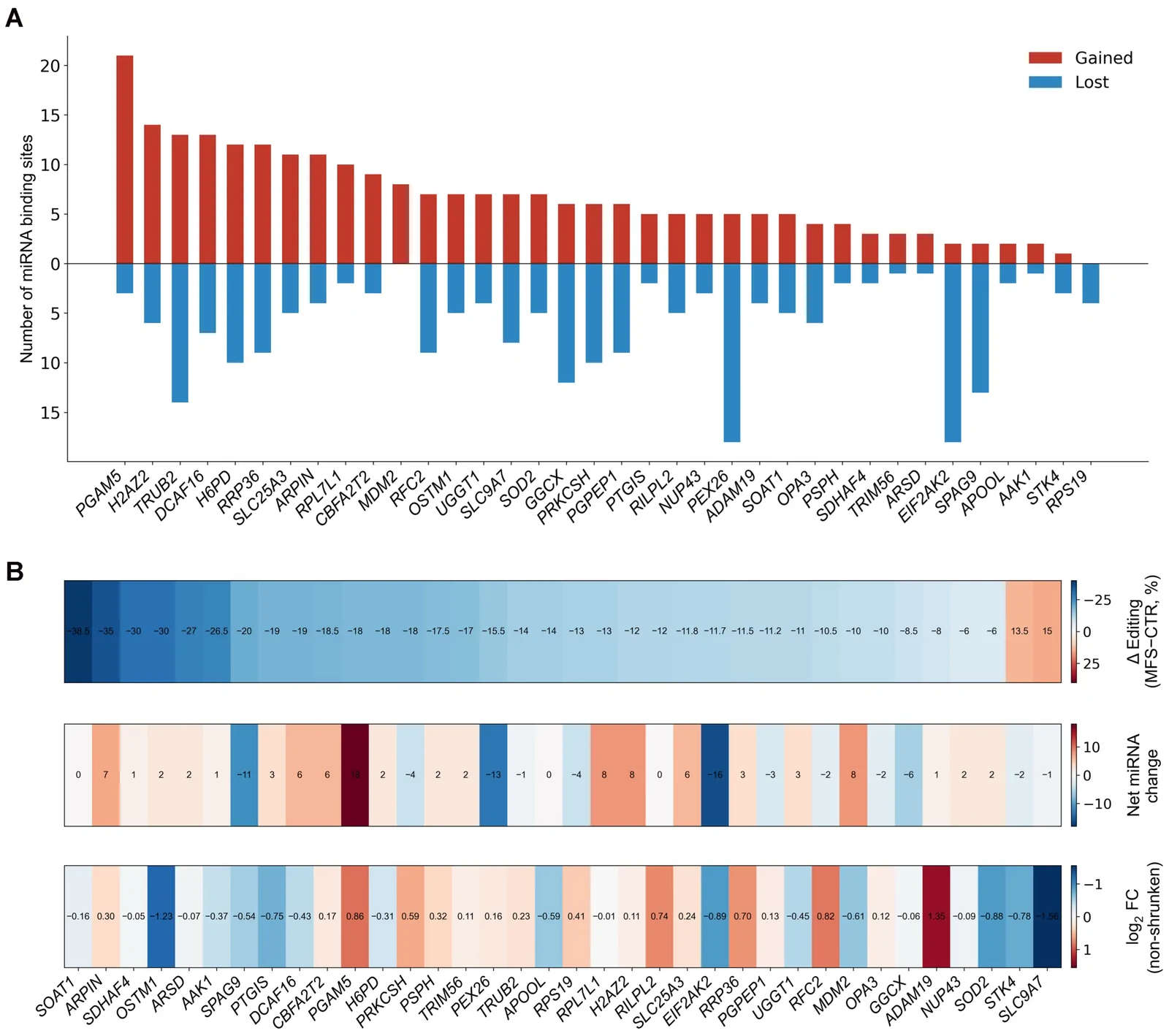
Integrated analysis of A-to-I RNA editing, miRNA targetome remodeling, and gene expression changes in MFS-derived vSMCs. (**A**) Number of miRNA binding sites gained (red) or lost (blue) as a consequence of A-to-I editing at the 47 significant 3′UTR editing sites across 36 target genes. miRNA target site prediction was performed by comparing the unedited (A) and edited (G) sequence variants within a ±50 nt genomic window using miRanda. Genes are ordered by net miRNA change (gained minus lost), from highest net gain to highest net loss. (**B**) Integrated heatmap displaying three layers of regulation for the 36 target genes, ordered by the magnitude of the editing change (Δ Editing, MFS−CTR, %) from most hypoedited (left) to most hyperedited (right). Top row: Δ Editing (%) between MFS and CTR vSMCs; negative values indicate hypoediting in MFS. Middle row: net miRNA site change (sites gained minus sites lost in the edited sequence); positive values indicate a net gain of miRNA binding sites upon editing. Bottom row: non-shrunken log_2_ fold change (MFS vs. CTR) from DESeq2 analysis of vSMC RNA-seq data; non-shrunken estimates were used to preserve directional information for genes not reaching statistical significance. Of 36 genes, 14 (39%) showed mRNA expression changes directionally concordant with the predicted editing-dependent miRNA retargeting effect.

To systematically assess the impact of RNA editing on miRNA-mediated regulation, we performed miRNA target site prediction for all 47 significant 3′UTR editing sites, comparing the unedited (A) and edited (G) sequence variants within a ±50 nt genomic window. Across 36 target genes, a total of 243 miRNA binding sites were gained and 215 were lost as a consequence of editing. The majority of genes (21 of 36) showed a net gain of miRNA target sites in the edited sequence, while 12 showed a net loss and 3 were neutral (Figure 5A, Supplementary Table S6). Since editing is globally reduced in MFS, the unedited adenosine form prevails, meaning that miRNA sites present only in the edited sequence are effectively lost in MFS, while sites present only in the unedited sequence are gained. The genes with the largest net gain of miRNA sites in the edited sequence, and therefore the largest functional loss of miRNA targeting in MFS, were *PGAM5* (net gain: +18; ΔE = −18%), *H2AZ2* (+8; ΔE = −12%), *RPL7L1* (+8; ΔE = −13%), *MDM2* (+8; ΔE = −10%), and *ARPIN* (+7; ΔE = −35%). Conversely, the genes with the largest net loss of miRNA sites in the edited sequence, and thus a net gain of miRNA repression in MFS, included *EIF2AK2* (net change: −16; ΔE = −11.7%), *PEX26* (−13; ΔE = −15.5%), and *SPAG9* (−11; ΔE = −20%) (Figure 5A, Supplementary Table S6).

To assess whether the predicted changes in miRNA targeting translate into concordant gene expression effects, we applied a site-specific concordance analysis integrating the direction of miRNA site changes (gained vs. lost in MFS), the direction of the editing change (hypoediting in MFS), and the observed mRNA fold change from DESeq2 (Figure 5B, Supplementary Table S6). Non-shrunken log2 fold changes were used to preserve the direction and magnitude of expression differences, as apeglm shrinkage collapses non-significant fold changes toward zero and would obscure directional trends for this analysis. Under the hypothesis that hypoediting reduces miRNA-mediated repression, genes with a net gain of miRNA sites in the edited sequence are expected to show higher mRNA levels in MFS (i.e., positive log2FC), while genes with a net loss of edited miRNA sites are expected to show lower mRNA levels. Of the 36 genes analysed, only 14 (39%) showed expression changes directionally concordant with the predicted miRNA retargeting effect, a proportion below the 50% expected by chance alone (Figure 5B, Supplementary Table S6). Concordant genes included *PGAM5, H2AZ2, RRP36, SLC25A3, ARPIN, CBFA2T2, ADAM19, PSPH,* and *TRIM56* (net miRNA gain in edited sequence, upregulated in MFS), and *SLC9A7, GGCX, EIF2AK2, SPAG9,* and *STK4* (net miRNA loss or hyperediting, downregulated in MFS). The remaining 19 genes (53%) showed expression changes opposite to predictions, and none of the 36 genes reached statistical significance after multiple testing correction (all padj > 0.05) (Figure 4B, Supplementary Table S5). The below-chance concordance rate indicates that editing-dependent miRNA retargeting at these 3′UTR sites does not systematically drive mRNA-level changes in MFS vSMCs, suggesting that the functional impact of this regulatory layer may operate primarily at the level of translational efficiency, act in a context-dependent manner, or be masked by compensatory post-transcriptional mechanisms.

Collectively, these findings support a model in which A-to-I RNA editing at 3′UTR sites constitutes an additional, largely underexplored layer of post-transcriptional gene regulation in the MFS aortic wall. The predominantly hypoedited landscape, paradoxically co-occurring with ADAR enzyme upregulation, suggests that increased ADAR expression in MFS may represent a reactive response to aortic wall stress rather than a driver of increased editing activity at specific 3′UTR sites. Validation in larger cohorts and through site-specific functional assays will be essential to formally establish the causal relationship between 3′UTR editing and gene expression at these loci.

## 3. Discussion

This study provides, to our knowledge, the first systematic characterization of ADAR1 and ADAR2 protein expression across the three major etiological subtypes of human TAA, MFS, BAV-associated TAA and TAV-associated TAA. MFS specimens exhibited the highest expression levels of both ADAR1 and ADAR2, with particularly pronounced ADAR2 upregulation in the endothelial compartment (median 51.0% in MFS-TAV vs. 23.6% in BAV and 19.1% in TAV; *p* < 0.001). This pattern is biologically plausible; the FBN1-mutation-dependent dysregulation of TGF-β signaling and the resulting chronic pro-inflammatory environment in the MFS aortic wall, characterized by elevated IFN-γ and PDGF-BB, are well-established inducers of ADAR1 transcription [28,45], and the most severely affected aneurysmal context would be expected to produce the greatest ADAR induction. ADAR2 upregulation in MFS is also notable given that ADAR2 is physiologically highly expressed in healthy vascular tissue, where it edits substrates such as Filamin A and IGFBP7 to maintain vascular homeostasis [31,33]; its further upregulation in the aneurysmal wall may represent a reactive response aimed at sustaining these homeostatic functions under conditions of progressive structural degeneration. Correlation analysis between ADAR expression and patients’ clinical parameters revealed a distinct and cell-type-specific pattern. In BAV and TAV patients, no significant association was found between ADAR1 or ADAR2 expression and classical cardiovascular risk factors, hypertension, smoking, or diabetes, in either ECs or vSMCs (all *p* > 0.05; Supplementary Table S1). This independence from conventional vascular risk factors suggests that ADAR upregulation in the aneurysmal wall reflects a disease-intrinsic molecular alteration rather than a secondary consequence of systemic comorbidities and supports its potential relevance as a tissue marker of aortic pathology per se. Notably, no significant correlation was found between ADAR expression and EuroSCORE II across any patient subgroup, indicating that ADAR upregulation is not simply a surrogate of overall surgical risk or systemic frailty. In the MFS-TAV subgroup, a sex-related difference was observed, with ADAR1 expression in vSMCs significantly higher in male compared to female patients (*p* = 0.029), raising the possibility of a sex-dependent modulation of ADAR1 activity in the smooth muscle compartment in the specific genetic context of MFS, a finding that warrants confirmation in larger cohorts. Spearman correlation analysis between ADAR expression and aortic dimensions revealed two distinct patterns. ADAR2 expression in vSMCs correlated positively with ascending aorta diameter in MFS patients, and a parallel positive correlation between ADAR2 expression and aorta dimensions was observed in TAV, indicating that ADAR2 upregulation tracks with the degree of structural aortic dilation across genetically distinct forms of TAA. Conversely, ADAR1 expression in vSMCs showed a significant negative correlation with the sinotubular junction dimension in MFSpatients, a divergent pattern that suggests a mechanistically distinct role for ADAR1 in the smooth muscle compartment of the MFS aortic wall. Together, these data establish ADAR2 as a molecular correlate of aortic dilation severity across TAA subtypes and identify a context-specific, inverse relationship between ADAR1 activity in vSMCs and sinotubular junction remodeling in MFS, pointing to cell-type-specific and isoform-specific functions of RNA editing enzymes in the structurally compromised aortic wall. This apparent divergence, ADAR1 being most strongly induced in MFS as a group yet inversely associated with sinotubular dilation within the MFS subgroup itself, may reflect two distinct biological phenomena rather than a single contradictory effect. At the group level, the pronounced ADAR1 upregulation in MFS likely reflects the intensity of the FBN1-mutation-driven TGF-β and inflammatory stimulus, which exceeds that of BAV- or TAV-associated TAA. Within the MFS subgroup, however, ADAR1 levels may instead track the adequacy of the vSMC compensatory response to this same stimulus, with patients mounting a more robust response better preserving aortic wall integrity, and an insufficient response permitting greater structural deterioration. Alternatively, or in addition, higher vSMC ADAR1 could simply mark a more transcriptionally intact, less degenerated vSMC population, since cystic medial necrosis involves progressive vSMC loss and dedifferentiation that could secondarily lower measurable ADAR1 in more severely affected specimens, independent of any active protective function. Notably, the editing-independent mechanism by which ADAR1 stabilizes MMP2 and MMP9 mRNAs via HuR, which would predict a positive rather than inverse relationship with severity, has so far been demonstrated in abdominal aortic aneurysm models [29] and not in TAA or in MFS-derived vSMCs; this pathogenic axis may therefore not be the dominant ADAR1-dependent mechanism operating in the MFS aortic wall. A further, more speculative possibility is that standard immunohistochemistry does not distinguish the constitutive ADAR1-p110 isoform from the interferon-inducible p150 isoform, and a shift in isoform composition with disease severity could also contribute to this pattern; this would require isoform-specific detection methods to test directly. These interpretations remain speculative and warrant dedicated mechanistic and longitudinal investigation in MFS-derived vSMCs. Given that MFS patients exhibited the most pronounced ADAR dysregulation, and that ADAR-dimension correlations were most clearly defined in the vSMC compartment of the aortic media, we extended the analysis to the transcriptomic and RNA editing landscape of MFS-derived aortic vSMCs using publicly available RNA-sequencing data (GSE128101; n = 3 MFS, n = 3 controls). This dataset, derived from isolated vSMC cultures rather than whole aortic tissue, provides a cell-type-specific view of MFS aortopathy, free from the confounding effects of cellular heterogeneity inherent to bulk tissue preparations.

Transcriptomic profiling of MFS-derived vSMCs identified 30 differentially expressed genes, of which 18 carried biologically interpretable fold changes. The upregulated signature was consistent with a shift toward a pro-inflammatory and synthetic vSMC phenotype, involving mediators of TNF superfamily signaling, vascular inflammation, and putative T lymphocyte-vSMC crosstalk. Interestingly, *ALDH1A2*, a key enzyme in retinoic acid biosynthesis, was among the most upregulated genes, pointing to a potentially novel role of retinoid signaling in vSMC dedifferentiation in MFS. Conversely, the downregulated gene set included regulators of receptor tyrosine kinase signaling and cytoskeletal dynamics whose suppression may further compromise contractile vSMC homeostasis in the remodeling aortic wall. GSEA against the MSigDB Hallmark collection revealed upregulation of cell cycle, inflammatory, and phenotypic plasticity programs, consistent with multi-directional vSMC transdifferentiation [16,20]. Downregulation of interferon response gene sets, with ADAR1 in their leading edge, most likely reflects loss of the in vivo inflammatory milieu in cell culture rather than true suppression of ADAR1 activity.

RNA editing analysis of the GSE128101 dataset identified 77 A-to-I editing sites reaching statistical significance, distributed across multiple genomic regions, 47 in 3′UTRs, 14 in ncRNA intronic, 7 in ncRNA exonic, 4 in coding exons, and 5 in intronic regions. The predominance of 3′UTR sites is consistent with the known enrichment of ADAR substrates in inverted Alu repeat structures within this genomic context [34,35].

Notably, among the 77 significant sites, four were located within protein-coding exons: *PPIL3* (p.S59G), *SLC12A6* (p.H1141R), *CTSB* (p.R31G/R268G), and *VHL* (p.Q124Q), and all four were hypoedited in MFS, indicating a global reduction in A-to-I recoding at these loci. These are the only sites in the dataset capable of directly altering the encoded protein sequence or codon usage and thus represent the most functionally consequential editing events identified. The *PPIL3* editing site (p.S59G) has been previously catalogued among conserved A-to-I recoding events in human cardiovascular tissues [53], underscoring its biological relevance. The *CTSB* recoding site is embedded in an ADAR1-dependent autoregulatory feedback loop, whereby S-nitrosylation of cathepsin B protein promotes ADAR1 recruitment to its own mRNA via the ADD1/MATR3 axis, stabilizing the transcript [55]; the hypoediting of *CTSB* observed in MFS vSMCs may disrupt this loop, with consequences for cathepsin B mRNA stability, protein expression, and ECM remodeling capacity. The *SLC12A6* (p.H1141R) and *VHL* (p.Q124Q) editing events were not detected in current public RNA editing catalogues and may represent novel recoding sites specific to vascular smooth muscle contexts. Given the roles of KCC3 (*SLC12A6*) in vSMC volume regulation and ion homeostasis, and of VHL in HIF-1α-dependent hypoxia sensing, the reduced frequency of these edited isoforms in MFS vSMCs warrants dedicated functional investigation.

Strikingly, 73 of the 77 significant sites (95%) showed reduced editing in MFS compared to controls. This predominantly hypoedited landscape, co-occurring with ADAR1 and ADAR2 protein upregulation, constitutes an apparent paradox. To investigate the enzymatic basis of site-specific editing changes, we performed per-sample Pearson correlation analysis between ADAR1 or ADAR2 mRNA levels and editing levels at each of the 77 significant sites individually. *ADAR1* mRNA showed statistically significant positive correlations (*p* < 0.05) with 19 of the 77 sites, with the strongest associations observed for *RRP36* site 2 (r = +0.980), *RBM3* (r = +0.970), *PSPH* (r = +0.960), and *SLC25A3* site 2 (r = +0.955). Three sites showed significant negative *ADAR1* correlations, *STK4* (r = −0.928), *VOPP1* (r = −0.903), and *SLC25A3* site 1 (r = −0.826), all corresponding to sites with increased editing in MFS, identifying ADAR1 as the predominant driver of the site-specific editing landscape in MFS vSMCs. Importantly, the direction of these correlations, whereby higher *ADAR1* mRNA associates with greater editing activity at 19 of 77 sites and lower ADAR1 with reduced editing, is directionally consistent with the clinical observation that MFS patients with more advanced ascending aortic dilation exhibit lower ADAR1 expression in vSMCs. The three sites showing significant negative *ADAR1* correlations (*STK4, VOPP1, SLC25A3* site 1) correspond exclusively to hyperedited loci, reinforcing a coherent model in which ADAR1 activity tracks editing level bidirectionally across the significant site landscape. However, because these correlations are derived from only six RNA-sequencing samples split evenly between MFS and controls, they may largely reflect this binary case–control grouping rather than a genuine within-disease severity gradient. The apparent convergence between the transcript-level editing correlations in the GSE128101 dataset and the protein-dimension correlations in our 58-patient cohort should therefore be regarded as hypothesis-generating rather than confirmatory cross-level evidence that reduced ADAR1 catalytic activity in vSMCs is a molecular correlate of disease severity in MFS. In contrast, *ADAR2* mRNA showed no statistically significant correlations with editing at any of the 77 sites, indicating that ADAR2, despite its protein-level upregulation in MFS aortic tissue, does not appear to drive site-specific editing activity in this cellular context. Downstream of the editing changes, systematic miRNA seed-match analysis of the 47 significantly differentially edited 3′UTR sites predicted widespread remodeling of the miRNA targetome, with editing-dependent changes affecting 458 predicted miRNA-mRNA interactions across 36 target genes. Since the vast majority of significant editing sites are hypoedited in MFS, 3′UTR sequences predominantly retain the unedited adenosine state, shifting the regulatory balance toward miRNA sites that recognize the unedited form while reducing engagement of those requiring the edited inosine. The genes with the largest net gain of miRNA sites in the edited sequence, and therefore the largest functional loss of miRNA targeting in MFS, were *PGAM5* (net gain: +18), *H2AZ2* (+8), *RPL7L1* (+8), *MDM2* (+8), and *ARPIN* (+7), while those with the largest net loss included *EIF2AK2* (−16), *PEX26* (−13), and *SPAG9* (−11). To assess whether this predicted remodeling translates into concordant gene expression changes, we integrated the direction of miRNA site gain or loss with observed mRNA fold changes from DESeq2, using non-shrunken log2 fold changes to preserve directional information. Only 14 of 36 genes (39%) showed directionally concordant expression changes, a proportion below the 50% expected by chance, indicating that editing-dependent miRNA retargeting at these 3′UTR sites does not systematically drive mRNA-level changes in MFS vSMCs, consistent with the known predominance of translational repression over mRNA destabilization for most miRNA seed-match types.

Taken together, these results support a model in which hypoediting of 3′UTR sites constitutes a broad epitranscriptomic remodeling of the miRNA targetome that likely exerts its primary functional effects at the translational rather than transcript level. The findings of this study have direct implications for the clinical management of TAA and MFS-associated aortopathy. Risk stratification in this field still relies predominantly on aortic diameter thresholds that are well recognized as insufficient to capture the biological heterogeneity of disease progression. A substantial proportion of dissections and ruptures occur in patients below the surgical threshold [7], highlighting the urgent unmet need for molecular markers capable of complementing anatomical assessment. Our data identify ADAR2 expression in vSMCs as a consistent correlate of ascending aortic diameter across genetically distinct TAA subtypes, MFS, and TAV-associated, suggesting that ADAR2 quantification in surgically obtained aortic specimens may provide prognostic information regarding the degree of structural dilation independently of cardiovascular comorbidities. The divergent behavior of ADAR1, which shows an inverse correlation with the sinotubular junction dimension in MFS, further indicates that ADAR1 and ADAR2 track different aspects of aortic remodeling and may have complementary rather than redundant biomarker potential. At the epitranscriptomic level, the 22 *ADAR1*-correlated editing sites identified here, 19 positively correlated (including *RRP36* site 2, *RBM3, PSPH, SLC25A3* site 2, and *TRUB2* site 1) and three negatively correlated sites (*STK4, VOPP1, SLC25A3* site 1), represent a novel class of candidate molecular markers whose systematic quantification in larger, prospective cohorts could open new avenues for monitoring ADAR1-dependent epitranscriptomic activity in MFS patients. Notably, among the four exonic recoding sites identified, *SLC12A6* was the only one showing a significant positive correlation with *ADAR1* mRNA (r = +0.883, *p* = 0.047), suggesting that ADAR1 may directly drive the A-to-I recoding event at this locus and that its hypoediting in MFS reflects reduced ADAR1 catalytic activity at a site with direct amino acid consequences.

This study should be interpreted in light of the limitations inherent to integrative analyses of rare human aortic material. Most notably, ADAR protein quantification and A-to-I editing profiling were performed in two independent cohorts that differed in demographic composition and sample preparation. Despite repeated attempts to extract RNA from residual frozen tissue of the immunohistochemistry cohort for a matched validation, the markedly fibrotic nature of this material yielded RNA of insufficient quantity and quality for reliable downstream analyses; optimization of extraction protocols for this tissue type is ongoing. Moreover, because tissue samples were collected at a single time point during surgery, the observed associations cannot establish whether ADAR dysregulation precedes or follows aortic remodeling, or whether it predicts subsequent clinical outcomes. The limited availability of non-aneurysmal aortic tissue and the small RNA-sequencing dataset further warrant validation of these findings in larger independent cohorts. Non-aneurysmal thoracic aortic tissue is exceptionally difficult to obtain for research purposes, autopsy specimens typically already show evidence of an ongoing degenerative process by the time of collection, while retrieval of a thoracic aortic segment from an organ donor is a rare occurrence even in the absence of active cardiac pathology, which explains why only a single control specimen could be obtained over the entire collection period. The editing and correlation analyses should therefore be viewed as a strategy for prioritizing candidate loci rather than as evidence of direct enzyme-substrate relationships. Moreover, editing-dependent changes in the miRNA-binding landscape were inferred computationally and were not complemented by direct measurements of miRNA expression, target engagement, or protein abundance. Because neither ADAR activity nor individual editing sites were experimentally perturbed, the proposed regulatory relationships should be regarded as testable hypotheses requiring functional validation. To directly address these limitations, we plan to establish primary aortic vSMC cultures from newly recruited MFS patients, enabling RNA extraction, RNA-sequencing, and protein extraction to be performed in parallel on the same specimens, including Western blot analysis of the top candidate genes emerging from the miRNA retargeting analysis (e.g., *PGAM5, EIF2AK2, MDM2, ARPIN*). Nevertheless, by integrating human tissue pathology with cell-type-specific transcriptomic analyses, this study provides an initial epitranscriptomic characterization of MFS-associated aortopathy, identifies ADAR1 and ADAR2 as candidate tissue biomarkers of this dysregulation, and nominates a focused set of ADAR1-associated exonic and 3′ UTR editing sites for functional validation as potential modulators of thoracic aortic wall remodeling.

## 4. Materials and Methods

### 4.1. Patient Population and Tissue Samples

This study included 58 patients with thoracic aortic aneurysm (TAA) undergoing surgical repair of the ascending aorta at the Cardiac Surgery Unit of the University of Rome Tor Vergata between July 2017 and June 2023, including 18 patients with MFS Syndrome (16 with a tricuspid and 2 with a bicuspid aortic valve, referred to as MFS-TAV and MFS-BAV respectively), 19 with tricuspid aortic valve (TAV)-associated TAA, and 21 with bicuspid aortic valve (BAV)-associated TAA. One healthy aortic specimen, without aortic pathology, was obtained from organ donor through the transplantation center of the University of Tor Vergata. The study was approved by the local institutional review board (Protocol title: 01-Aorta-2018; Protocol number: 179/18), and all participants provided written informed consent. Patient data were anonymized to ensure confidentiality.

### 4.2. Immunohistochemistry and Histopathological Analysis

Immunohistochemical analyses were performed on residual aortic tissue samples in the Anatomic Pathology Department of Tor Vergata University Hospital. Aortic samples were fixed in 10% formalin for 24 h and embedded in paraffin. Serial 4-micrometer sections were deparaffinized, subjected to antigen retrieval in citrate buffer (pH 6.0, 98 °C, 30 min), and blocked with goat serum. Sections were incubated overnight at 4 °C with mouse monoclonal anti-ADAR1 and anti-ADAR2 antibodies (1:100; Santa Cruz Biotechnology, Dallas, TX, USA), followed by biotinylated secondary antibody and streptavidin-peroxidase. Staining was developed with DAB chromogen and counterstained with hematoxylin. ADAR1 staining intensity was scored on a 0–3 scale (0 = negative, 1 = weak, 2 = moderate, 3 = strong); for ADAR2, the percentage of positive cells per field (×200 magnification) was calculated. Positive and negative controls were included in all experiments.

### 4.3. Transcriptomic and RNA Editing Analysis

Publicly available paired-end RNA-seq data from primary aortic vSMCs derived from individuals with MFS and healthy controls (SRP188087 from GSE128101) were retrieved from the Sequence Read Archive. In the original study, total RNA was extracted from human aortic SMC cultures using Trizol Reagent (Life Technologies, Carlsbad, CA, USA) and purified with the miRNeasy Mini kit (Qiagen S.A, Hilden, Germany.) according to the manufacturer’s instructions, with DNase digestion performed for each sample; RNA concentration was estimated by absorbance at 260 nm and RNA quality was assessed by agarose gel electrophoresis [47]. Raw reads were quality filtered and adapter trimmed using fastp, removing low-quality reads and retaining sequences with a minimum length of 55 nucleotides. Processed reads were aligned to the GRCh38 reference genome using STAR in two-pass mode and GENCODE gene annotations, allowing only uniquely mapped reads and a maximum mismatch rate of 4%. PCR duplicates were marked with Picard software version number 3.3.0, and library strandedness was verified using RSeQC software version number **5.0.3**. Gene and transcript abundance was quantified using featureCounts software version number 2.0.6 and StringTie software version number 2.2.3, respectively. Differential gene-expression analysis between MFS and control vSMCs was performed with DESeq2 after retaining genes with at least 10 counts in two or more samples. Control samples were used as the reference group, log_2_ fold changes were shrunk using the *apeglm* method, and genes with a Benjamini–Hochberg-adjusted *p* value < 0.05 and |log_2_ fold change| ≥ 1 were considered differentially expressed. Variance-stabilized expression values were used for sample-distance analysis and heatmap generation, while pathway enrichment was investigated through over-representation analysis and gene set enrichment analysis using Gene Ontology Biological Process and MSigDB Hallmark collections. RNA-editing candidates were independently detected using REDItools software version number 2.0 and JACUSA2 software version number 2.0.2 and retained through a strand-aware filtering workflow requiring adequate read coverage and support for canonical A-to-I-compatible substitutions. Known polymorphisms were excluded using dbSNP 151 annotations, whereas potentially artefactual supporting reads were identified through pBLAT software version number 2.5.1 remapping and by detecting reads containing multiple non-canonical mismatches. Splice-junction information obtained with RegTools software version number 1.0.0 was also incorporated to reduce alignment-related artefacts near exon boundaries. The final candidate sites were functionally annotated with ANNOVAR (https://annovar.openbioinformatics.org, accessed on 2 March 2025) according to the GENCODE Comprehensive Gene Annotation release 50 (GENCODE CompV50), and their editing levels were quantified across all samples for differential comparison between MFS and control cells. StringTie-derived transcript abundances were additionally used to verify whether the assigned exonic, UTR or nonsynonymous consequences were compatible with the isoforms effectively expressed in the analysed samples.

### 4.4. miRNA Target Site Prediction

For each of the 47 significant 3′UTR editing sites (*p* < 0.05, |ΔMedian| ≥ 10%), a ±50 nt genomic window was retrieved from hg38 via the UCSC Genome Browser REST API. Two sequence variants were generated: unedited (A) and edited (G). miRNA seed-based prediction was performed against 2,656 human mature miRNAs from miRBase v22, using canonical seed match types (6mer, 7mer-1a, 7mer-m8, 8mer) following the TargetScan framework [57]. miRNAs were classified as ‘gained’ (match only in G-form), ‘lost’ (match only in A-form), or ‘shared’. For each gene, a net miRNA change score (gained minus lost) was calculated. Results were visualized as bar charts and integrated heatmaps. All analyses were performed in Python 3.9 using pandas software version number 2.2.3, numpy software version number 1.26.4, matplotlib software version number 3.9.4, seaborn software version number 0.13.2, biopython software version number 1.84, and requests software version number 2.32.3.

### 4.5. Statistical Analysis

For immunohistochemical and clinical data, all experiments were repeated at least three times. Data are presented as mean ± SD. Normality was assessed by the D’Agostino & Pearson test (alpha = 0.05). For comparisons of two groups, Student’s unpaired two-tailed *t*-test was used for normally distributed data; Mann–Whitney two-tailed test for non-normally distributed data or groups with n < 7. For more than two groups, one-way ANOVA with Tukey post-hoc test was used for normally distributed data; Kruskal–Wallis with Dunn’s multiple comparisons test for non-normally distributed data. Analyses were performed in Prism 9.0 (GraphPad Software, San Diego, CA, USA) or RStudio (v1.4.1717). Differences were considered statistically significant at *p* < 0.05. Correlation analyses between ADAR expression and clinical/pathological variables were exploratory and were not corrected for multiple comparisons.

### 4.6. Correlation Analyses Between ADAR Expression, RNA Editing, and Gene Expression

To determine whether the A-to-I editing at significant editing sites is preferentially driven by ADAR1 or ADAR2, Pearson correlation analysis was performed between per-sample ADAR1 and ADAR2 mRNA expression levels (DESeq2 normalized counts) and the editing level (E %) at each of the 77 significant editing sites across the six individual RNA-sequencing samples (n = 3 MFS, n = 3 CTR). For each site, two separate Pearson r coefficients were computed (one for ADAR1, one for ADAR2) with two-tailed *p*-values; a site was considered significantly correlated with an ADAR enzyme at *p* < 0.05. Results were visualized as a heatmap of Pearson r values and as scatter plots for significant sites. To further assess whether editing-level changes at significant editing sites could directly affect mRNA stability independently of miRNA-mediated regulation, a second Pearson correlation analysis was performed between the per-sample editing level (E, %) and the per-sample normalized expression counts (DESeq2 software version number 1.44.0) of the host gene, across the six samples, for each of the 77 significant editing sites. A positive correlation would indicate co-occurrence of higher editing with higher transcript abundance, whereas a negative correlation would suggest editing-associated transcript destabilization. All correlation analyses were performed in Python 3.9 using scipy.stats.pearsonr.

### 4.7. Concordance Analysis Between miRNA Retargeting and Gene Expression

To assess whether the editing-dependent changes in predicted miRNA target sites translate into concordant gene expression effects, a site-specific concordance analysis was performed for each of the 36 genes harboring significant 3′UTR editing sites. For each gene, the direction of the net miRNA change (positive = net gain of miRNA binding sites in the edited G-form; negative = net loss) was compared with the direction of the observed mRNA fold change in MFS versus CTR. Under the hypothesis that hypoediting reduces miRNA-mediated repression, genes with a net gain of miRNA sites in the edited sequence are predicted to show higher mRNA levels in MFS (positive log2FC), while genes with a net loss of edited miRNA sites are predicted to show lower mRNA levels (negative log2FC). Non-shrunken log2 fold changes from DESeq2 were used for this analysis, as apeglm shrinkage collapses non-significant fold changes toward zero, which would systematically obscure directional trends for genes not reaching statistical significance. Non-shrunken log2FC values were computed from DESeq2 normalized counts as log2[(mean_MFS + 0.5)/(mean_CTR + 0.5)], where 0.5 is a pseudocount added to avoid log of zero. A gene was classified as concordant if its log2FC direction matched the prediction, discordant if opposite, or neutral if net miRNA change was zero. The proportion of concordant genes was compared to the 50% expected by chance. All analyses were performed in Python 3.9 using pandas and numpy.

## Data Availability

The original contributions presented in this study are included in the article/Supplementary Materials. Further inquiries can be directed to the corresponding author.

## Supplementary Materials

The following supporting information can be downloaded at https://www.mdpi.com/article/10.3390/ijms27188250/s1.
