# Supplementary material for: ADAR1 and ADAR2 Expression in the Thoracic Aortic Wall Correlates with Aneurysm Severity and Dissection Risk: Insights into A-to-I RNA Editing Dysregulation in Marfan Syndrome-Derived vSMCs
Source: Int J Mol Sci. 2026 Sep 16;27(18):8250. doi: 10.3390/ijms27188250 (PMC13607941; doi:10.3390/ijms27188250)
Supplement: Supplementary file 1 [file ijms-27-08250-s001.zip › Supplementary Table S1.pdf]

Supplementary Table S1. Demographic and clinical characteristics by patient group.

Variable	BAV (n=21)	TAV (n=19)	MARFAN-TAV (n=16)	MARFAN-BAV (n=2)	MARFAN TOT (n=18)	Overall p-value †	Effect on ADAR (within group) ‡
**Demographics**							
Age (years) — median (IQR)	65.0 (58.0–68.0)	70.0 (68.0–72.5)	30.0 (26.0–51.5)	52.0 (40.0–64.0)	30.0 (28.0–58.5)	**<0.001**	BAV: p=0.039* TAV: n.s. MFN-TAV: n.s. MFN TOT: n.s.
Male sex — n (%)	19/21 (90%)	15/19 (79%)	9/16 (56%)	2/2 (100%)	11/18 (61%)	0.318	BAV: n.s. TAV: n.s. MFN-TAV: p=0.029* MFN TOT: n.s.
Hypertension — n (%)	13/21 (62%)	18/19 (95%)	15/16 (94%)	2/2 (100%)	17/18 (94%)	0.992	BAV: n.s. TAV: n.t. MFN-TAV: n.t. MFN TOT: n.t.
Smoking (current/past) — n (%)	13/21 (62%)	13/19 (68%)	16/16 (100%)	2/2 (100%)	18/18 (100%)	**0.002**	BAV: n.s. TAV: n.s. MFN-TAV: n.t. MFN TOT: n.t.
Diabetes — n (%)	2/21 (10%)	5/19 (26%)	15/16 (94%)	2/2 (100%)	17/18 (94%)	**<0.001**	BAV: n.s. TAV: n.s. MFN-TAV: n.t. MFN TOT: n.t.
Aortic valve dysfunction — n (%)	20/21 (95%)	19/19 (100%)	16/16 (100%)	2/2 (100%)	18/18 (100%)	—	BAV: n.t. TAV: n.t. MFN-TAV: n.t. MFN TOT: n.t.
**Aortic dimensions**							
Aortic bulb (mm) — median (IQR)	42.0 (40.0–45.0)	48.0 (40.0–53.5)	48.0 (45.0–49.2)	45.5 (44.8–46.2)	47.0 (45.0–49.0)	0.052	BAV: n.s. TAV: n.s. MFN-TAV: n.s. MFN TOT: n.s.
Sinotubular junction (mm) — median (IQR)	36.0 (31.0–42.0)	42.0 (31.5–49.5)	44.0 (36.0–50.0)	42.0 (38.0–46.0)	44.0 (36.0–50.0)	0.069	BAV: n.s. TAV: p=0.042* MFN-TAV: p=0.016* MFN TOT: p=0.036*
Ascending aorta (mm) — median (IQR)	49.0 (49.0–51.0)	51.0 (48.0–53.5)	48.0 (43.8–53.8)	48.0 (46.0–50.0)	48.0 (44.0–52.0)	0.652	BAV: n.s. TAV: p=0.020* MFN-TAV: p=0.048* MFN TOT: p=0.022*
**Operative risk**							
EuroSCORE II (%) — median (IQR)	4.6 (3.8–7.5)	5.3 (4.0–8.6)	4.3 (3.8–7.5)	2.7 (2.2–3.2)	3.9 (3.8–6.7)	0.300	BAV: n.s. TAV: n.s. MFN-TAV: n.s. MFN TOT: n.s.
**ADAR expression**							
ADAR1 endothelium — median (IQR)	1.8 (1.5–2.2)	2.1 (1.7–2.4)	2.7 (2.5–2.7)	2.2 (2.1–2.4)	2.6 (2.5–2.7)	**<0.001**	BAV: p<0.001* TAV: p=0.011* MFN-TAV: p=0.021* MFN TOT: p=0.004*
ADAR1 SMC — median (IQR)	1.8 (1.6–2.3)	2.0 (1.9–2.3)	2.7 (2.4–2.9)	2.2 (2.1–2.3)	2.6 (2.4–2.8)	**<0.001**	BAV: p<0.001* TAV: p=0.011* MFN-TAV: p=0.021* MFN TOT: p=0.004*
ADAR2 endothelium (%) — median (IQR)	23.6 (15.5–32.0)	19.1 (16.2–27.6)	51.0 (37.5–65.9)	36.5 (31.1–42.0)	47.9 (30.3–62.5)	**<0.001**	BAV: p<0.001* TAV: p=0.040* MFN-TAV: p<0.001* MFN TOT: p=0.009*
ADAR2 SMC (%) — median (IQR)	13.5 (7.4–21.0)	15.8 (12.7–20.6)	24.4 (15.8–27.9)	25.8 (19.9–31.7)	24.4 (15.7–27.9)	**0.018**	BAV: p<0.001* TAV: p=0.048* MFN-TAV: p<0.001* MFN TOT: p=0.009*

† Between-group comparison. Continuous variables: Kruskal–Wallis test. Categorical variables: chi-square test (MARFAN-BAV excluded due to n=2). Smoking includes current and past smokers. Significant p-values (p<0.05) in bold red. ‡ Within-group association with ADAR expression. Continuous variables: Spearman correlation; categorical variables: Mann–Whitney U test (e.g. male vs female, YES vs NO). For ADAR expression rows,

*correlation with the other three ADAR variables is reported (self-correlation excluded). Minimum p-value across ADAR variables shown. * $p<0.05$; n.s. = not significant; n.t. = not testable. BAV, bicuspid aortic valve; TAV, tricuspid aortic valve; MFN-TAV, Marfan syndrome with tricuspid aortic valve; MFN TOT, all Marfan patients; IQR, interquartile range; SMC, smooth muscle cells.*
