# Supplementary material for: ADAR1 and ADAR2 Expression in the Thoracic Aortic Wall Correlates with Aneurysm Severity and Dissection Risk: Insights into A-to-I RNA Editing Dysregulation in Marfan Syndrome-Derived vSMCs
Source: Int J Mol Sci. 2026 Sep 16;27(18):8250. doi: 10.3390/ijms27188250 (PMC13607941; doi:10.3390/ijms27188250)
Supplement: Supplementary file 1 [file ijms-27-08250-s001.zip › Supplementary Table S2.pdf]

Supplementary Table S2. Correlations between ADAR expression and aortic clinical parameters. Spearman rho coefficients with listwise deletion. * $p < 0.05$; ** $p < 0.01$; *** $p < 0.001$. Statistically significant correlations in bold. ADAR1 expression scored 0–3 (IHC intensity); ADAR2 as % positive cells/field.

ADAR protein	Clinical variable	BAV (n=21)	TAV (n=19)	MARFAN-TAV (n=16)	MARFAN TOT (n=18)
**ADAR1 ECs**	Age	r=-0.13 p=0.583	r=-0.14 p=0.558	r=0.22 p=0.412	r=0.12 p=0.626
	Aortic bulb (mm)	r=-0.04 p=0.862	r=0.26 p=0.276	r=-0.08 p=0.757	r=0.09 p=0.728
	Sinotubular junction (mm)	r=-0.16 p=0.491	r=0.40 p=0.093	r=-0.14 p=0.599	r=-0.14 p=0.588
	Ascending aorta (mm)	r=0.36 p=0.104	r=-0.10 p=0.677	r=0.02 p=0.952	r=-0.03 p=0.909
	EuroSCORE II (%)	r=-0.32 p=0.160	r=-0.25 p=0.307	r=-0.07 p=0.793	r=0.08 p=0.749
**ADAR1 vSMCs**	Age	r=0.19 p=0.402	r=0.01 p=0.980	r=-0.03 p=0.908	r=-0.08 p=0.749
	Aortic bulb (mm)	r=-0.08 p=0.732	r=0.29 p=0.227	r=-0.38 p=0.143	r=-0.20 p=0.433
	Sinotubular junction (mm)	r=-0.20 p=0.380	r=0.28 p=0.243	**r=-0.59 p=0.016**	**r=-0.50 p=0.036**
	Ascending aorta (mm)	r=0.39 p=0.080	r=-0.13 p=0.586	r=-0.38 p=0.150	r=-0.36 p=0.148
	EuroSCORE II (%)	r=-0.02 p=0.946	r=0.01 p=0.963	r=0.23 p=0.401	r=0.31 p=0.205
**ADAR2 ECs**	Age	r=0.32 p=0.164	r=0.14 p=0.576	r=0.23 p=0.385	r=0.15 p=0.542
	Aortic bulb (mm)	r=0.28 p=0.212	r=0.16 p=0.524	r=0.00 p=0.996	r=0.07 p=0.792
	Sinotubular junction (mm)	r=0.22 p=0.337	r=0.37 p=0.124	r=0.40 p=0.130	r=0.29 p=0.237
	Ascending aorta (mm)	r=0.17 p=0.462	**r=0.53 p=0.020**	r=0.49 p=0.053	r=0.44 p=0.071
	EuroSCORE II (%)	r=0.05 p=0.834	r=-0.19 p=0.428	r=-0.12 p=0.670	r=-0.08 p=0.756
**ADAR2 vSMCs**	Age	**r=0.45 p=0.039**	r=0.06 p=0.820	r=0.02 p=0.943	r=0.17 p=0.488
	Aortic bulb (mm)	r=0.28 p=0.225	r=0.27 p=0.269	r=0.35 p=0.178	r=0.16 p=0.518
	Sinotubular junction (mm)	r=0.24 p=0.300	**r=0.47 p=0.042**	r=0.29 p=0.271	r=0.40 p=0.102
	Ascending aorta (mm)	r=0.09 p=0.703	r=0.06 p=0.821	**r=0.50 p=0.048**	**r=0.54 p=0.022**
	EuroSCORE II (%)	r=0.01 p=0.962	r=-0.07 p=0.772	r=-0.18 p=0.500	r=-0.10 p=0.679
